# The Network Structure of Adolescent Well-Being Traits: Results From a Large-Scale Chinese Sample

**DOI:** 10.3389/fpsyg.2019.02783

**Published:** 2019-12-20

**Authors:** Guang Zeng, Kaiping Peng, Chuan-Peng Hu

**Affiliations:** ^1^Department of Psychology, Sun Yat-sen University, Guangzhou, China; ^2^Department of Psychology, Tsinghua University, Beijing, China; ^3^German Resilience Center, Mainz, Germany

**Keywords:** mental health, well-being, adolescents, network analysis, measurement

## Abstract

**Background:** The mental health and well-being of adolescents are becoming increasingly important globally. Understanding the relationship between different aspects of well-being is crucial for effective interventions of the well-being of adolescents. The present study aims to analyze the network structure of adolescent well-being and identify the central well-being traits.

**Methods:** We used a network model to analyze the network structure of a psychometrically sound measurement of adolescent well-being – the engagement, perseverance, optimism, connectedness, and happiness (EPOCH) scale. The dataset comes from a representative sample of Chinese adolescents (17, 854 participants from rural and urban areas from Southern, Northern, and the middle part of China).

**Results:** The 20 items of EPOCH formed a highly interconnected network. The item H4 (“I am a cheerful person.”), item E2 (“I get completely absorbed in what I am doing”), and item O4 (“I believe that things will work out, no matter how diffcult they seem”) were the traits with the highest centrality in the network.

**Conclusions:** Cheerfulness, engagement in current activity, and optimism for the future are most central to the psychological well-being of Chinese adolescents. Future studies should further test the dynamics between these central traits and other well-being traits to find effective interventions of well-being of adolescents.

## Introduction

Mental health of adolescents is a global concern (Lopez et al., 2006). As mental health problem and well-being are likely to be two ends of a continuum (Caspi et al., 2014; Stochl et al., 2015), improving the well-being of adolescents may also help to reduce their mental health problems (Patel et al., 2018).

The primary step in improving adolescents’ well-being is understanding adolescents’ well-being itself. Keyes (2002) proposed that mental health is a syndrome of well-being symptoms, and mental health is created when a person exhibits high levels of hedonia and eudaimonia symptoms, such as positive functioning in social and emotional life (Keyes, 2009). According to World Health Organization, well-being is defined as “a state in which every individual realizes his or her own potential, can cope with the normal stresses of life, can work productively and fruitfully, and is able to make a contribution to her or his community” (World Health Organization, 2004). Ryff (1989) identifies the six components of well-being are autonomy, environmental mastery, positive relationships with others, purpose in life, realization of potential, and self-acceptance. Seligman (2011) proposes the PERMA model of well-being, which states that the fve basic elements of well being are Positive Emotion, Engagement, Relationships, Meaning, and Accomplishment. In particular for young people, Kern et al. (2016) proposed the EPOCH model to characterize the well-being of adolescents. This model is comprised of five clusters of positive characteristics: (1) engagement, the capacity to become absorbed in and focused on what one is doing; (2) perseverance, the ability to work hard and pursue one’s goals to the end even when facing obstacles; (3) optimism, i.e., hopefulness and confidence about the future; (4) connectedness, the sense of having a satisfying relationship with others and provide emotional support to others; (5) happiness, being generally happy, fun loving, and content with one’s life. Even though the EPOCH model was well supported by the questionnaire (Kern et al., 2016, 2018), the relationship of different aspects of these well-being traits are still unknown.

Network analysis can help to address this issue. Network analysis is a novel conceptual framework that has gained increasing attention in recent years in psychiatry and clinical psychology (Cramer et al., 2010; McNally et al., 2011; Schmittmann et al., 2013; Mõttus and Allerhand, 2018). In network analysis, each psychological construct (symptom, attitude, behavioral, traits, belief, etc.) is treated as a node, and the relationship between each pair of nodes is the edge that links those two nodes. By putting all items into a network, the network analysis can estimate which items are most “central,” i.e., have the most robust relationship with other items. The central items are likely to spread activation through the network once activated, whereas less important items with fewer connections lie on the periphery of a network (Fried et al., 2017). In the recent years, the network approach has been utilized in psychopathology and has been applied to a wide variety of disorders, including depression (van Borkulo et al., 2015), PTSD (Fried et al., 2018), and eating disorder (Smith et al., 2018).

Recently, applying the network analysis to data of the Warwick-Edinburgh Mental Well-being Scale (WEMWBS) from four-cohort data in the UK, Stochl et al. (2018) found that the most central items in the well-being network were related to positive self-perception and mood. These results suggested that positive self-perception and mood might be the optimal targets for the improvement of the mental well-being of adolescents of adolescents.

Given that mental health and well-being are strongly impacted by culture and social factors (e.g. Swartz, 1998; Marsella and Yamada, 2000; Kramer et al., 2002), the results from Stochl et al. (2018) might not be applied to other populations, such as Chinese. To further investigate the generalizability and cross-cultural variation of the central features of well-being, the current study extended Stochl et al. (2018) in two aspects: (1) we used a representative dataset of Chinese adolescents to examine the generalizability on the central features of well-being in an Eastern culture; (2) we used a different scale of well-being, which is a five-dimensional construct, instead of the one-dimensional structure of WEMWBS. More specifically, we applied network model to the 20-item Chinese version of EPOCH, which measures five clusters of well-being: engagement, perseverance, optimism, connectedness, and happiness (Kern et al., 2016, 2018). This questionnaire showed satisfying reliability and validity to measure the well-being of adolescents across different cultures (Kern et al., 2016, 2018). We used state-of-the-art network modeling techniques to identify the central items of well-being traits, assessing through the adolescents’ well-being measure (EPOCH) in a representative dataset gathered from 17,854 adolescents in 11 different samples from urban and rural areas, in Southern, Northern, and middle part of China.

## Methods

### Participants

We re-analyzed the data reported in previous studies, which came from 11 samples of adolescents from primary and secondary schools in rural and urban areas of Southern, Northern, and middle part of China, resulting in a total sample of 17,854 (9,548 males, 8,306 females, aged from 6 to 18). Besides, 5,459 students (2,827 males, 2,632 females) completed the EPOCH items a second time, between 3 and 16 months later, providing some indications of cross-time stability.

The system used to collect data only recorded complete responses, such that it is unknown how many students might have started the questionnaire and not completed it, or how many students within classes refused to participate.

The original data collection was conducted following the guideline of the Declaration of Helsinki and reviewed and approved by ethics committees and institutional review boards of Tsinghua University. All participants and their parents were informed about the objectives of the study and assured that all responses would be kept confidential, only accessible to the research group, and used for research purposes. All participants and their parents provided written informed consent before participation. Further ethical approval was not required for the current secondary data analysis.

Sample 1 included 778 students (382 males, 396 females) in grades 1 through 12 from one primary school and one junior high school in Chengdu City, Sichuan province (an urban area in Southwestern China). Sample 2 included 1,737 students (913 males, 824 females) from one primary school and one secondary school in Tianjin City (an urban area in Northern China). Sample 3 included 1,664 (891 males, 773 females) primary school students from Yiyang City, Hunan province (a rural area in Southern China). Sample 4 included 2,129 students (1,254 males, 875 females) from a vocational school in Yuncheng City, Shanxi province (rural area in central China). Sample 5 included 1,340 students (820 males, 520 females) from a vocational school in Hunan province (a rural area in Southern China). Sample 6 included 1,322 primary and secondary students (688 males, 634 females) from the Sichuan province (a rural area in Southwestern China). Students ranged from grade 2 in primary school to grade 3 in secondary school. Sample 7 included 2,493 students (1,339 males, 1,154 females) from one primary school, one technical secondary school, and one secondary school in the Hunan province (a rural area in Southern China). Sample 8 included 2,271 students (1,129 males, 1,142 females) from 35 primary and secondary schools in Chengdu City, Sichuan province (an urban area in Southwestern China). Sample 9 included 2,607 students (1,346 males, 1,261 females) from 35 primary and secondary schools in Chengdu City in Sichuan province (an urban area in Southwestern China). Sample 10 included 1,279 students (660 males, 619 females) from 10 primary and secondary schools in Chengdu City in Sichuan province (an urban area in Southwestern China). Sample 11 included 234 students (126 males, 108 females) from one primary school in Chengdu City in Sichuan province (an urban area in Southwestern China).

All participants finished EPOCH and other questionnaires as well; see Zeng and Kern (2019) for the procedure of data collection. Only the EPOCH data were analyzed and reported here.

### Measures

#### The Epoch Measure of Adolescent Well-Being

The adolescents’ well-being measure EPOCH includes 20 items, which measure five domains: Engagement, Perseverance, Optimism, Connectedness, and Happiness. Items are scored on a 1 to 5 scale (*not at all like me = 1; very much like me = 5*). Overall well-being is measured as the average of the scores of five domains. We adopted the Chinese version of EPOCH (Kern et al., 2018), which has been demonstrated as an adequate psychometric instrument.

### Network Analysis

We estimated a Gaussian graphical model (GGM) using the score of 20 items of EPOCH (ordinal data, form 1 ~ 5). The Gaussian graphical model (GGM) is a regularized partial correlation network to model the interaction between different components or constructs. In this graph, each item of EPOCH is depicted as circles, called “nodes” (or “vertices”). Nodes are connected by lines, called “edges.” The edges in GGM can be understood as conditional dependence relations among items: if two items are connected in the resulting network, they are dependent after adjusting for all other items. If no edge exists between two items, they are conditionally independent. The graphical LASSO (least absolute shrinkage and selection operator) was applied to estimate GGM (Epskamp and Fried, 2018) to avoid spurious edges, therefore, leading to a sparse network that describes data parsimoniously. To visualize the network, we used the Fruchterman-Reingold algorithm, which determines the position of a node based on the sum of connections it has with other nodes (Fruchterman and Reingold, 1991). However, the spatial closeness in the graph should not be over interpreted (Jones et al., 2018).

To identify the central items of well-being, we estimated the centrality of the EPOCH network. The centrality of a network can be measured in three different ways: betweenness, closeness, and node strength (Boccaletti et al., 2006). *Betweenness* can be understood as the relative frequency of a node of interest is in the shortest path between other node pairs. *Closeness* measures the sum of the shortest paths from the node of interest to all other nodes in the whole network (Opsahl et al., 2010). *Node strength* is the sum of the interrelation values (e.g., regularized partial correlation) of the node of interest with all nodes directly related to it (McNally, 2016; Costantini et al., 2019). The previous simulation suggested that betweenness and closeness may be not reliably estimated (Epskamp et al., 2018). Therefore, we focus on the node strength while reporting the estimates of betweenness and closeness in the Supplementary Figure S2.

To complement the centrality, we also calculated the *node predictability* as in Haslbeck and Fried (2017). Node predictability is the proportion of variance of a node that can be explained by all nodes linked with it. In this way, node predictability estimates the absolute measure of its interconnectedness (Epskamp et al., 2018).

Note that network stability was an issue that needs to be addressed in psychological network analysis (Forbes et al., 2017; Epskamp and Fried, 2018). Followed suggestions of previous study (Epskamp et al., 2017), we used 2000 bootstraps in the current study. The edge-weight accuracy was estimated by calculating the 95% confidence intervals of all edge weights. The stability of centrality was indexed by a centrality-stability coefficient (CS-coefficient), which should not be lower than 0.25 and preferably above 0.5 (Briganti et al., 2018). The *difference Test* function was used to test the edge-weights and centrality.

Finally, the test–retest reliability of the network was tested by comparing the network estimated from test and retest data, respectively. We formally tested the difference between these two networks *via* the R package *Network Comparison Test* (NCT) (van Borkulo et al., 2017, submitted). This method started with an omnibus test for each pair of the network to investigate whether all edges were identical, which was followed by *post hoc* tests to quantify how many of the edges differed across each pair of networks.

All analyses were carried out in R (version 3.5.2) in Rstudio 1.1.383. The package we used included qgraph, version 1.4.4 (Epskamp et al., 2012) and glasso (Friedman et al., 2014) for network estimation and visualization; mgm, version 1.2–2 for node predictability (Haslbeck and Waldorp, 2015); igraph, version 1.1.2 (Csárdi and Nepusz, 2014) for the spinglass algorithm; Exploratory Graph Analysis (Golino and Epskamp, 2017) for the walktrap algorithm; and bootnet, version 1.0.1 for stability (Epskamp and Fried, 2018). See the *sessionInfo* in the Supplementary Material for detailed information about the packages used in the current analysis (see, https://osf.io/9ts76/).

## Results

### The Well-Being Network

**Figure 1:**
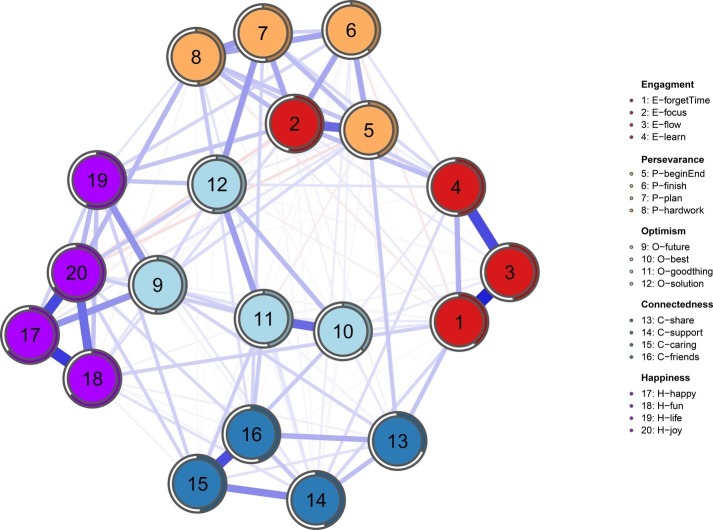
The network composed of the 20 items of EPOCH measure. Each item is represented by a node (1 to 20), and it belongs to a different community of well-being, indicated by a code in the column on the right: Engagement, Perseverance, Optimism, Connectedness, and Happiness subscales. Blue lines are positive connections, and red lines are negative connections. The thickness of the line represents the connection strength. Colored areas in the rings surrounding the nodes represent the node predictability (percentage of variance of a given node explained by surrounding nodes).

Overall, most items within the network are positively associated (see Figure 1). Item 3 (E3: “I get so involved in activities that I forget about everything else”) is strongly correlated with item 1 (E1: “When I do an activity, I enjoy it so much that I lose track of time”) and item 4 (E4: “I finish whatever I begin”). Item 17 (C4: “I have friends that I really care about”) has a wide edge to item 18 (H1: “I feel happy”) and item 20 (H4: “I am a cheerful person”). Other strong edges include item 15 (C3: “There are people in my life who really care about me”) and item 16 (C4: “I have friends whom I really care about”), item 2 (E2: “I get completely absorbed in what I am doing”) and item 5 (P1: “I finish whatever I begin”).

As for the node predictability, the mean node predictability is 48.7%, which means that on average, 48.7% of the variance of each node is explained by its neighbors. We can know how well the given node can be predicted by the other nodes surrounding it assuming that all edges go to the node under investigation from its neighbors.

### Centrality of Items

**Figure 2:**
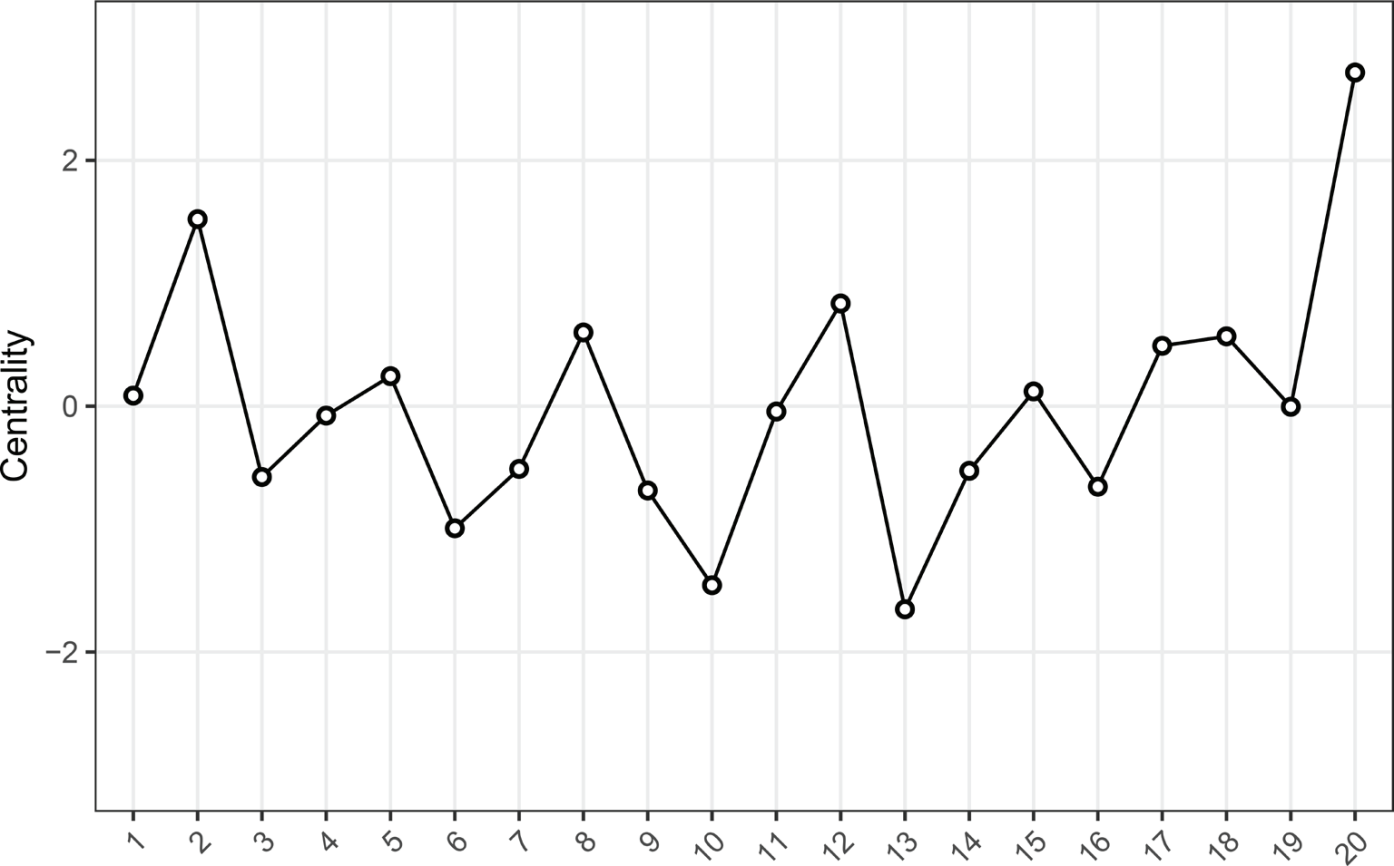
Strength centrality estimates for the 20-items EPOCH measure. The Y-axis represents the centrality indices as standardized z-scores (the greater the estimate, the more central the item is), and the X-axis represents the 20 EPOCH items.

Our centrality analysis revealed that item 20 (H4: “I am a cheerful person”) has the highest standardized strength centrality in the network as well as the highest node predictability (0.654, see Figure 2; see Supplementary Figure S2 for other centrality indices). Other central items include item 2 (E2: “I get completely absorbed in what I am doing”) and item 12 (O4: “I believe that things will work out, no matter how difficult they seem”). Item 13 (C1: “When something good happens to me, I have people whom I like to share the good news with”) and item 10 (O2: “In uncertain times, I expect the best”) represent the lowest strength centrality values.

### Network Accuracy and Stability

The bootstrap for edge-weight accuracy reveals relatively small CIs, which showed a more precise estimation (see Supplementary Figure S3). The edge-weight difference test shows that the well-being network is accurately estimated and that the most robust edges are significantly stronger than other edges (see Supplementary Figure S4).

As for the stability of centrality, our results reveal that CS coefficient values obtained are 0.67 for node betweenness, 0.67 for node closeness, and 0.75 for node strength (see Supplementary Figure S5). These results are above 0.5, suggesting that the centrality is stable (Epskamp and Fried, 2018). The centrality difference test shows that highest centrality estimates are statistically different from the lowest centrality estimates, even though a statistical difference is not shown among nodes with the highest strength centrality estimates (see Supplementary Figure S6).

Finally, we used Network Comparison Test (NCT) to compare the test–retest networks. The median value of *p* resulting from the permutation test of the maximum difference in edge weights (with 5,000 iterations) is 0.0526, and most values of *p* were between 0.04 and 0.08, suggesting these two networks might be significantly different. We further compared all individual edges, and 21 edges showed significant differences among 190 edges (see Supplementary Figure S1).

## Discussion

This study aimed to identify the central aspects of the psychological well-being of adolescents in a representative Chinese sample. We applied psychological network analysis to the well-being survey of a large and representative Chinese adolescent sample, in which the well-being was measured by a well-established psychological well-being measure developed for adolescents (EPOCH).

In general, our results revealed a closely related network between the items of EPOCH. Using a bootstrap approach, we found that the centrality of this well-being network is stable. Moreover, test–retest network comparison provided evidence that the well-being network has relative high stability and generalizability. Therefore, the network inference was warranted.

Importantly, we identified the items that showed the highest centrality, which might be the target that can maximize the effectiveness of future interventions. We used the *node strength* as the primary centrality index because of its stability. Our results revealed that the items with the highest centrality are item 20 (H4: “I am a cheerful person”), item 2 (E2: “I get completely absorbed in what I am doing”), and item 12 (O4: “I believe that things will work out, no matter how difficult they seem”). These results confirmed and extended previous studies. First, these results are consistent with the previous study, demonstrating that cheerfulness plays a central role in influencing other well-being traits in a large sample in the UK (Stochl et al., 2018). It suggests that cheerfulness is a central trait of well-being and this pattern seems to be cross-culture and cross-measurement stable. Second, these results revealed some important cultural differences as well. The relatively high stability (centrality) of item 2 (“completely absorbed in what I am doing”) and item 12 (“believe things will work out”) in our results suggest the importance of engagement and optimism in Chinese adolescents’ well-being.

The relative high centrality of item 2 (“completely absorbed in what I am doing”) might be explained by the vital role of academic performance for students in China (Zhang et al., 2007). It might be that engaging in current activities is essential for academic performance and therefore has strong influence on other aspects of adolescent’s well-being. The relative high centrality of item 12 is consistent with the previous study on the importance of optimism in resilience to stress (Kalisch et al., 2015, 2017). Note that the centrality indices also have high stability: all centrality stability coefficients are above the recommended criteria of 0.5 (Epskamp and Fried, 2018).

The current study found that the network constructed by test data and retest data might be different, which is consistent with the low reliability as revealed by traditional analysis of the same dataset (Kern et al., 2016; Zeng and Kern, 2019). The reasons behind the relatively low test-retest stability of the network structure may be the same as those contributed to the low test-retest reliability in the traditional analysis of this dataset (Kern et al., 2016; Zeng and Kern, 2019). Firstly, the lag between measurement occasions ranged from 3 to 16 months, which might be too long for adolescent (e.g., student might answer the questionnaire right before an examination for the test but after an examination for the retest). More importantly, many of the participated schools simultaneously implemented some positive education programs, which might change the well-being level of some students’ well-being.

## Strengths and Limitations

An important strength of the current study is that it utilized a large representative sample from both rural and urban areas from Northern, Southern, and middle part of China, supporting the generalizability of findings in Chinese culture (and East Asian more broadly). Furthermore, the sample was collected from different time points, which enabled us to validate the reliability to address the considerable concern about the replicability in the network literature (Forbes et al., 2017).

The identification of a group of highly connected traits of adolescents’ well-being has several practical implications. If it is acknowledged that certain traits are highly connected and central within a network, then these traits can be targeted in the adolescents’ well-being intervention plans. In the current study, three traits are most “central” in adolescents’ psychological well-being network: cheerfulness, absorbing in current activities, and optimism. In the literature, there are several adolescents’ well-being programs that have targeted the abovementioned central traits, such as Positive Psychology School Based Intervention (Shoshani and Steinmetz, 2014), Positive Education Program (Seligman et al., 2009), and Promoting Resilience In Stress Management (PRISM) (Rosenberg et al., 2015). The improvement of a trait with high centrality may largely influence the overall well-being network and and facilitated the well-being intervention gains. Additionally, future research should further identify which traits drive the course of adolescents’ well-being longitudinally.

The current study also has some limitations. First, as we mentioned in previous reports (Kern et al., 2018; Zeng and Kern, 2019), the data collection process has some limitations. For example, the computer software used to collect data in the current study only recorded complete responses. It is unknown how many respondents began the questionnaire but did not finish. Thus, the analyses essentially use a case-wise deletion approach, despite the many known drawbacks, with no way of estimating the extent to which missingness affected the results. Second, the network model analysis was based on a group level analysis. This means that network properties such as structure or centrality may not replicate in the same way in a single individual. Third, network analysis presents edge as a putative causal connection, assuming that nodes differ from each other meaningfully. If two nodes represent the same aspect of a psychological construct, an edge is not a putative causal connection but represents shared variance (Fried and Cramer, 2017). EPOCH measure in some cases has this problem. For example, item 3 (E3: “I get so involved in activities that I forget about everything else”) and item 2 (E2: “I get completely absorbed in what I am doing”) seem to measure the same construct. Therefore, future work might consider combining the latent variable model and network analysis (Epskamp et al., 2017). Last, the current study only used a single measure tool, the EPOCH measure, to index the psychological well-being of adolescents. Even though EPOCH is a well-established measure (Kern et al., 2016) and and its psychometrical properties has been tested with Chinese respondents (Zeng and Kern, 2019), there are other well-established measures. Future studies could employ other adolescents’ well-being measures to perform network analysis, and see whether the results are consistent.

## Conclusions

In conclusion, the current study reveals that three traits have the highest degree of centrality in adolescents’ psychological well-being network: (1) being cheerful (H4: “I am a cheerful person”); (2) being absorbed in current activities (E2: “I get completely absorbed in what I am doing”); (3) being optimistic and hopeful toward future (O4: “I believe that things will work out, no matter how difficult they seem”). These traits might serve as the targets of interventions to improve the psychological well-being of adolescents.

## Data Availability Statement

The datasets analyzed in this manuscript are not publicly available. Requests to access the datasets should be directed to zengg6@mail.sysu.edu.cn.

## Ethics Statement

The studies involving human participants were reviewed and approved by Tsinghua University Ethics Committee. Written informed consent to participate in this study was provided by the participants’ legal guardian/next of kin.

## Author Contributions

GZ and C-PH designed and carried out the experiments and wrote the manuscript. C-PH analyzed the experimental results. KP supervised the experiment and the analyzing and writing process.

### Conflict of Interest

The authors declare that the research was conducted in the absence of any commercial or financial relationships that could be construed as a potential conflict of interest.
