# Supplementary material for: The Network Structure of Adolescent Well-Being Traits: Results From a Large-Scale Chinese Sample
Source: Front Psychol. 2019 Dec 20;10:2783. doi: 10.3389/fpsyg.2019.02783 (PMC6932967; doi:10.3389/fpsyg.2019.02783)
Supplement: Supplementary file 1 [file Data_Sheet_1.docx]

**Supplementary materials for:**

**The network structure of adolescent well-being traits: Results from a Large-Scale Chinese sample**

Guang Zeng

Department of psychology, Sun Yet-sen University, Guangzhou, China

Kaiping Peng
Department of Psychology, Tsinghua University, Beijing, China

Chuan-Peng Hu *
German Resilience Center, Mainz, Germany

URL: https://www.frontiersin.org/articles/10.3389/fpsyg.2019.02783


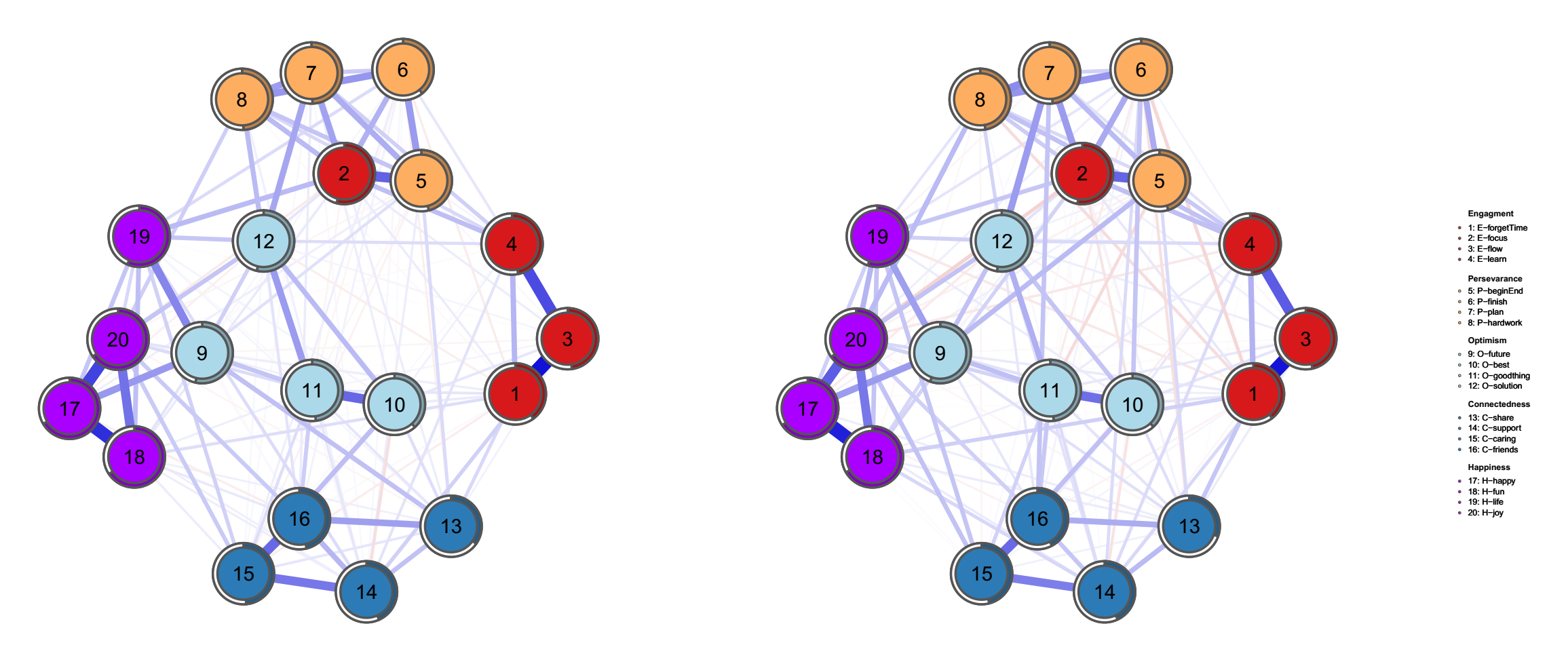


Figure S1. The network of network of 20 EPOCH items from the first test (left) and second test (right).


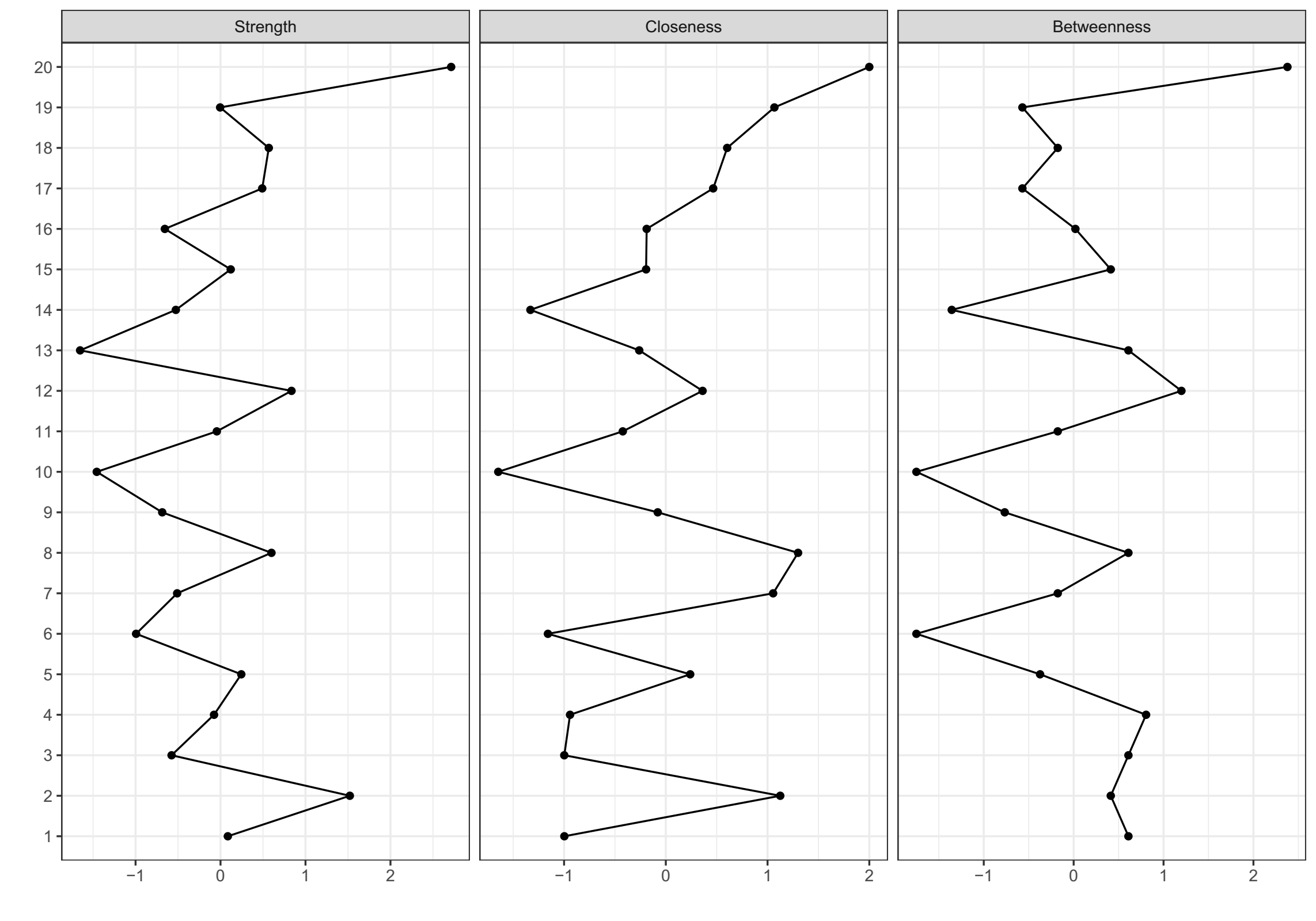


Figure S2. Strength, closeness and betweenness of EPOCH network.


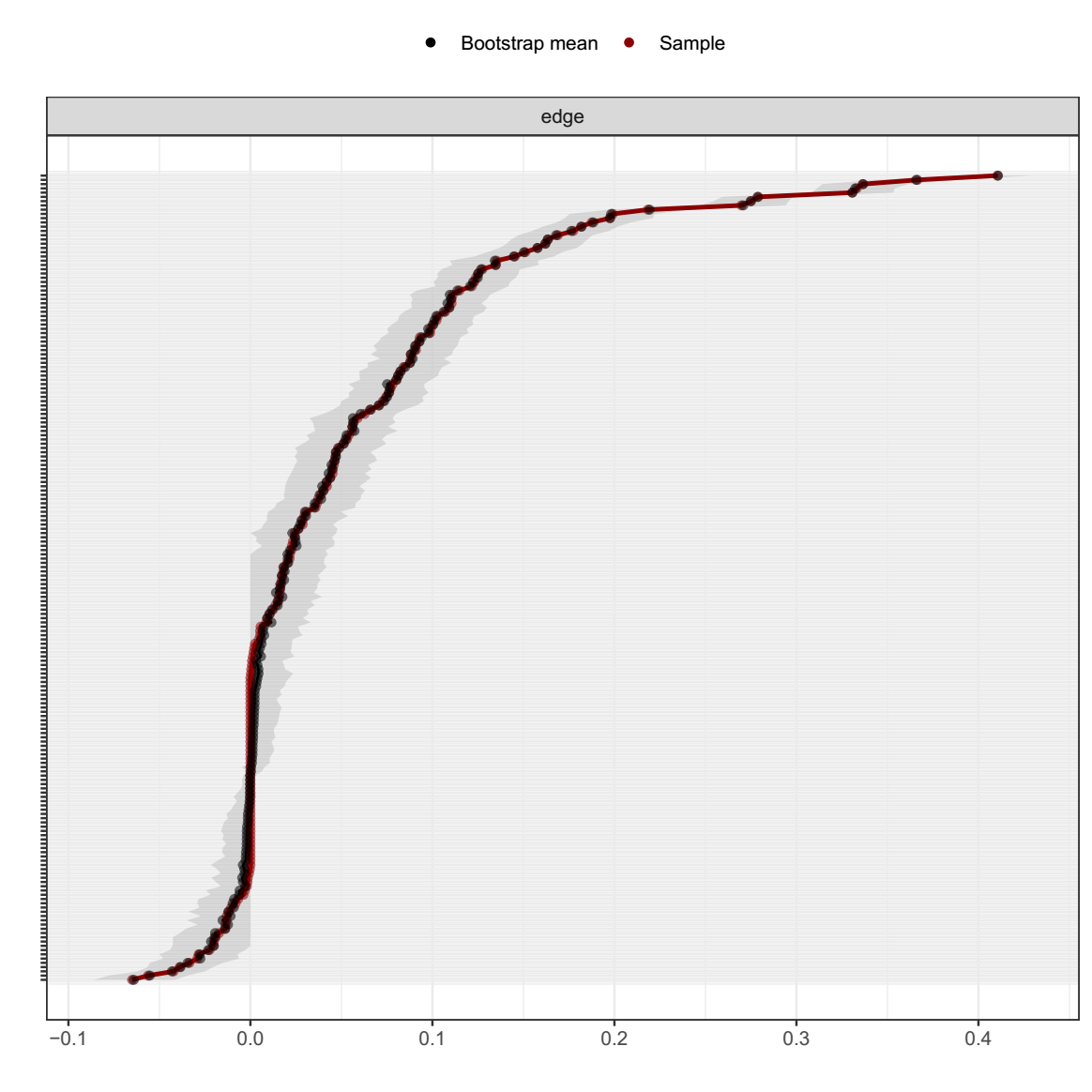


Figure 3. Bootstrapped confidence intervals of estimated edge-weights for the estimated network of 20 EPOCH items. The red line indicates the sample values and the gray area the bootstrapped CIs. Each horizontal line represents one edge of the network. The y-axis labels have been removed to avoid cluttering.


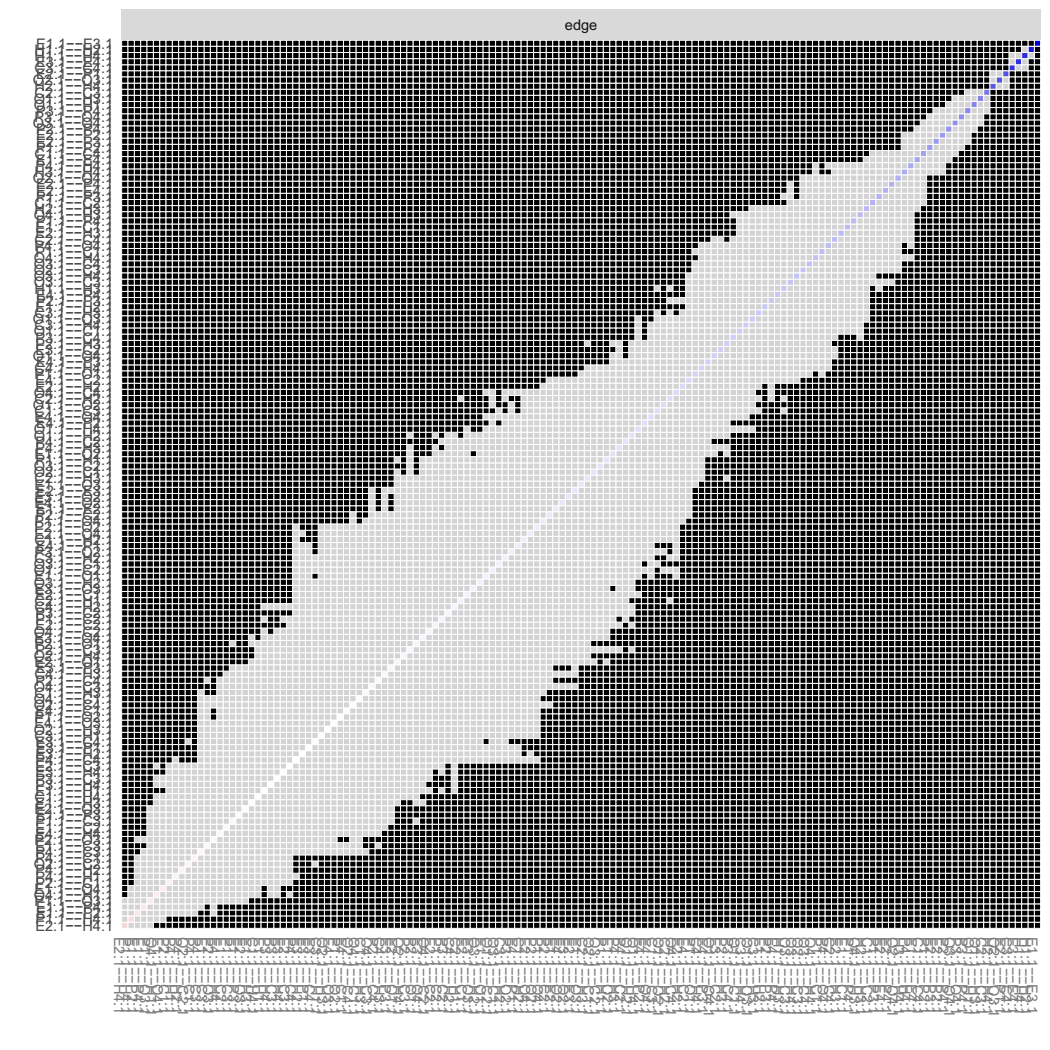


Figure S4. Bootstrapped difference tests (α = 0.05) between edgeweights that were non-zero in the estimated network. Gray boxes indicate edges that do not differ significantly from one-another and black boxes represent edges that do differ significantly from oneanother. Colored boxes in the edge-weight plot correspond to the color of the edge in Fig. 1 in the main text.


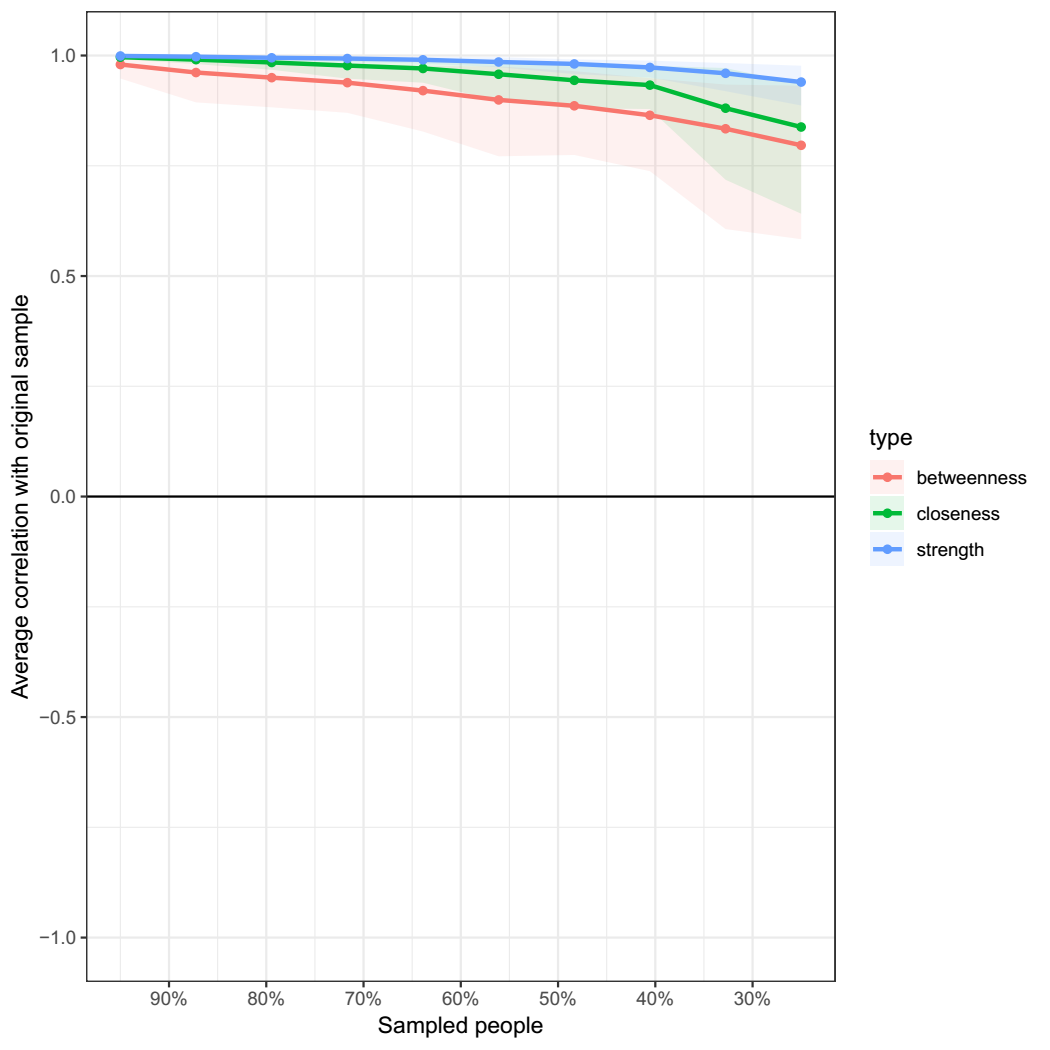


Figure S5. Average correlations between centrality indices of networks sampled with persons dropped and the original sample. Lines indicate the means and areas indicate the range from the 2.5th quantile to the 97.5th quantile.


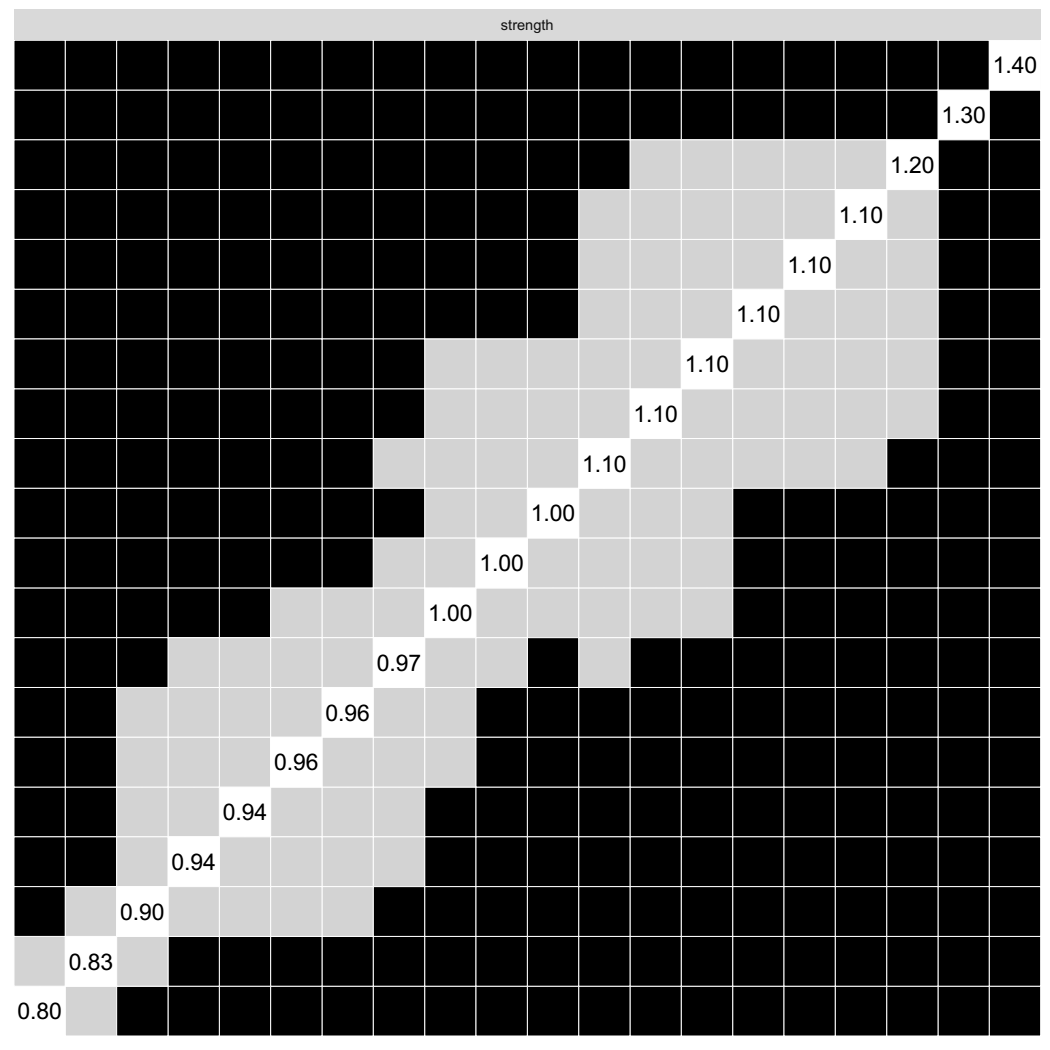


Figure S6. Bootstrapped difference tests (α = 0.05) between node strength of the 20 EPOCH items. Gray boxes indicate nodes that do not differ significantly from one-another and black boxes represent nodes that do differ significantly from oneanother. White boxes in the centrality plot show the value of node strength
